# OPM-based fetal magnetocardiography: fetal cardiac time intervals in healthy pregnancies compared to postnatal ECGs

**DOI:** 10.1007/s00404-026-08403-5

**Published:** 2026-03-23

**Authors:** Annette Wacker-Gussmann, Karin Narushima, Gabriela Tardelli, Ronald T. Wakai, Janette F. Strasburger, Lena Wunderl, Tobias Jensch, Reinhard Heckel, Silvia M. Lobmaier, Nicole Nagdyman, Peter Ewert, Peter Fierlinger

**Affiliations:** 1 Clinic of Congenital Heart Disease and Pediatric Cardiology, German Heart Center, TUM University Hospital, Technical University of Munich, Munich, Germany; 2 Department of Medical Physics, University of Wisconsin Madison, Madison, WI USA; 3 Departments of Pediatrics (Cardiology) and Biomedical Engineering Medical College of Wisconsin, Milwaukee, WI USA; 4 Chair E66, TUM School of Natural Sciences, Technical University of Munich, Munich, Germany; 5 Department of Computer Engineering, Information and Technology, TUM School of Computation, Technical University of Munich, Munich, Germany; 6 Department of Obstetrics and Gynecology, School of Medicine & Health, TUM University Hospital, Technical University of Munich, Munich, Germany

**Keywords:** Fetal magnetocardiography, OPM, SQUID, Norm values, New technology, Fetal cardiology

## Abstract

**Background:**

Fetal magnetocardiography (fMCG) is the most accurate method to assess fetal heart rhythm and conduction. New quantum sensor technology makes it possible to use less expensive devices. The aim of the study is to measure cardiac time intervals of healthy fetuses with a new technology, optically pumped magnetometry (OPM), and compare these results with conventional SQUID-based fMCG and postnatal ECGs.

**Methods:**

The recordings were made using an OPM-based fMCG system and a person-sized magnetic shield, established at German Heart Center,TUM University, Munich, Germany. The subjects were 57 healthy women with uncomplicated singleton pregnancies, studied at a mean gestational age of 32 ± 3.7 weeks with an overall range of 25–40 weeks. The P, PR, QRS, QT, QTc, and RR intervals were measured and compared with published data from previous fMCG devices and postnatal ECG.

**Results:**

The P, PR, and QRS intervals increased with gestational age, but the QT and QTc intervals did not. The measured values of the OPM device were consistent with those from previously published data SQUID values. U-waves were seen in 17.3% of subjects. Eleven subjects were studied by fMCG after 30 weeks’ gestation and by ECG within 17 weeks of birth. In this cohort, the P-wave duration, QRS duration, and QTc increased after birth, but the PR and QT intervals did not.

**Conclusion:**

The results obtained with our innovative OPM-based fMCG system are comparable to previously available measurements obtained by other technologies. The data establish prediction intervals for OPM-based fMCG waveforms in normal fetuses, which is essential for future clinical application. The technology can be used to recognize fetuses with rhythm or conduction abnormalities that might not be evident by echocardiography. To our knowledge, this is the first report comparing fetal cardiac time intervals measured by OPM-based fMCG with postnatal ECG. Lengthening of cardiac intervals consistent with increased chamber size was seen postnatally.

## What does this study adds to the clinical work

**Table:** 

The data establish prediction intervals for innovative OPM-based fMCG waveforms in normal fetuses, which is essential for future clinical application. The technology can be used to recognize fetuses with rhythm or conduction abnormalities that might not be evident by echocardiography.	

## Introduction

In clinical routine, fetal heart rhythm is mainly assessed using ultrasound-based cardiotocography and fetal echocardiography. Both methods measure the fetal electrical heart activity indirectly. Critical electrophysiological information about the fetal heart rhythm is often missing. The use of fetal electrocardiography (fECG) is limited due to the insulating properties of the vernix caseosa during the second and third trimester of pregnancy. Fetal magnetocardiography (fMCG) has been shown to overcome these problems [1].

FMCG is the magnetic analog of the fECG. The fMCG can precisely assess fetal heart rate patterns, heart rate reactivity, heart rhythm, and conduction. In recent decades, fetal magnetocardiography (fMCG) has developed into an innovative technology that provides insights into the electrophysiology of fetal arrhythmias that cannot be obtained with ultrasound. FMCG can be used to detect conduction and repolarization disorders. It has been shown that fMCG provides critical information in fetal congenital heart disease (CHD), cardiomyopathies, and fetuses at risk of inherited arrhythmia syndromes [1–5]. The efficacy of fMCG for clinical evaluation of serious fetal arrhythmia was affirmed in the Scientific Statements of the American Heart Association and Heart Rhythm Society [6, 7].

The primary factor limiting the widespread clinical adoption of fMCG has been the considerable cost and complexity associated with conventional superconducting quantum interference device (SQUID) technology. SQUIDs require complex cryogenics, consume large amounts of liquid helium, and must be operated in a large, expensive magnetically shielded room. However, recent advances in quantum sensor technology usher in a new era of technological innovation. Among the most promising developments are optically pumped magnetometers (OPM), which are much smaller, easier to use, and more cost-effective than traditional sensors. [8–10].

In this study, we used OPM sensors that are operated within an open and comfortable, person-sized shield designed by the Department of Physics (Chair E66) TUM Munich and used in the German Heart Center, TUM Munich, Germany. The aim of this study is to confirm the excellent technical capabilities of this new highly innovative system by comparing its data to that of SQUID systems and postnatal ECG.

## Methods

The study was performed by the Department of Congenital Heart Disease, German Heart Center, TUM University Hospital, TUM School of Medicine and Health and the Department of Physics Chair of Precision Measurements at Extreme Conditions, TUM School of Natural Sciences. The protocol for this prospective observational study was approved by our institutional review board. Participants were partly recruited from the Department of Obstetrics and Gynecology, TUM University Hospital, TUM School of Medicine and Health. Informed consent was obtained from all subjects.

### Subjects

The subjects were 57 healthy women with uncomplicated singleton pregnancies with a mean age of 32.1 years (SD ± 3.9 years), studied at 32 (SD ± 3.7) weeks’ gestation with a range from 25 to 40 weeks. Maternal BMI (Body Mass Index) was 21.9 kg/m^2^ (SD ± 4.2). The pregnancies were considered uncomplicated if the mothers were not assigned to a high-risk obstetrical team. Five pregnancies were conceived after assisted reproductive technologies. Measurements were performed in 57 subjects; five were excluded due to low signal-to-noise ratio or magnetic artifact. Each subject was studied once. Parents agreed to return once with their infant for postnatal ECG.

### Data acquisition

OPM system: The magnetic shield consists of a cylindrical, person-sized magnetic shield built from three layers. The tri-axial Magnetic Field Cancelling System MR-3 by Stefan Mayer Instruments Dinslaken, Germany, provides additional active field compensation, yielding an ambient noise floor of 80 fT/Hz^1/2^. The set of 16 QuSpin Zero Field Magnetometers (QZFMs) from QuSpin Inc., Louisville, CO, USA, is fixed in a flat 4 × 4 grid with 5 cm grid spacing below the abdomen. The OPM sensors are operated in dual-axis mode.

The mother changed into non-magnetic clothing and was positioned prone on the patient table. A brief ultrasound exam was performed to locate the fetal heart to guide probe placement. During each session, three runs of fMCG were recorded, each lasting 10 min or longer.

### Signal processing and averaging

Signal processing and data analysis were performed using methods developed by the Biomagnetism Lab at the University of Wisconsin–Madison [11] and summarized here. A digital filter with a 1–50 Hz passband was applied to band-limit the data. The sampling rate was 1 kHz.

Signal processing was used to remove the maternal MCG and other interferences. Specifically, Independent component analysis (ICA) was used to separate the fetal and maternal components, and the maternal component and other interferences were removed from the data [12]. We manually identified and templated the maternal QRS signals for extraction.

Using the QRS complexes as triggers, averaged waveforms were computed from 50 to 100 consecutive QRS complexes during periods when the fetal heart rate was at or near baseline. The heart rate was considered to be at baseline when the fetus was quiescent, and the heart rate was within 5 bpm of a stable minimum seen over the duration of the recording. Fetal quiescence was inferred from an absence of fetal movement, as indicated by fMCG actography tracings [13] displayed simultaneous with the fetal heart rate tracings.

### Waveform interval measurements

**Fig. 1:**
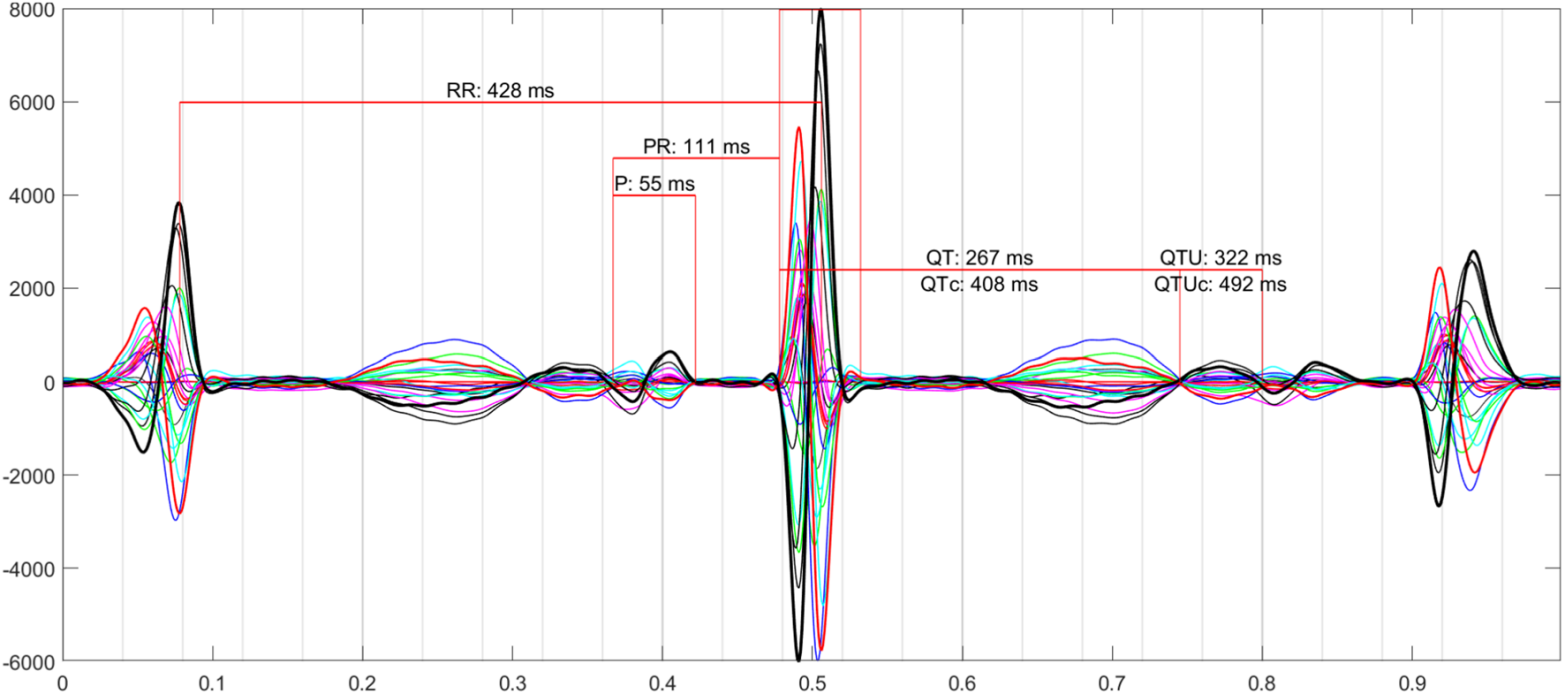
Example of a butterfly waveform plot showing fetal cardiac time interval measurements. A U-wave (arrow) is visible

Waveform intervals—P, PR, QRS, QT, QTc, and RR—were measured by a specialist trained in fMCG from “butterfly” plots (Fig. 1), which superimposed the signals from all channels and were verified by the authors (AW-G and KN).

#### Prenatal measurements

The P interval was measured from the beginning to the end of the P-wave. The PR interval was measured from the beginning of the P-wave to the beginning of the QRS complex and corresponds to the time from the onset of atrial depolarization to the onset of ventricular depolarization. The QRS interval was measured from the beginning to the end of the QRS complex and corresponds to the duration of ventricular depolarization. The QT interval was measured from the beginning of the QRS complex to the end of the T-wave and corresponds to the time from the beginning of ventricular depolarization to the end of ventricular repolarization. The RR interval was measured from the peak of the QRS complex to the peak of the next QRS complex and corresponds to the time between ventricular beats QTc was computed using Bazett’s formula: QTc = QT/RR^1/2^.

### Postnatal electrocardiogram

A standard 12-lead ECG (Schiller, Germany) with ten electrodes in the unsedated infant with ECG amplitude of 0.1 mV/mm and 25 mm sweep speed was done. Manual and electronic measurements were done by one physician blinded to the prenatal finding within the first year of life, as not all parents were able to come back immediately after birth.

### Statistical analysis

The statistical analysis of the clinical data collected was performed using MATLAB (The Mathworks, Inc., Natick, Massachusetts). Descriptive statistics (e.g., mean, standard deviation, and prediction intervals) were used for characterization. The correlation of the waveform intervals with gestational age was assessed using ordinary least-squares linear regression. For comparison, we used data from the SQUID study of Strand et al., which were made available to us, in addition to other published SQUID fMCG data.

The results of the respective cardiac time intervals pre- and postnatally were visualized in scatter plots. A paired *t*-test was used to determine whether the mean value of the cardiac time intervals before and after birth was significantly different. The residuals were evaluated for normality using the Shapiro–Wilk test. The analysis was restricted to subjects studied as fetuses after 30 weeks of gestation and as infants prior to age 17 weeks to focus on changes specifically associated with birth, while minimizing the confounding influence of gestational and postnatal age. Linear regression was used to verify that the fMCG and ECG data, respectively, did not show a dependence on gestational age or postnatal age over the age ranges used in the comparison. A significance level of *p* < 0.05 was considered statistically significant.

## Results

### Fetal cardiac time intervals

**Fig. 2:**
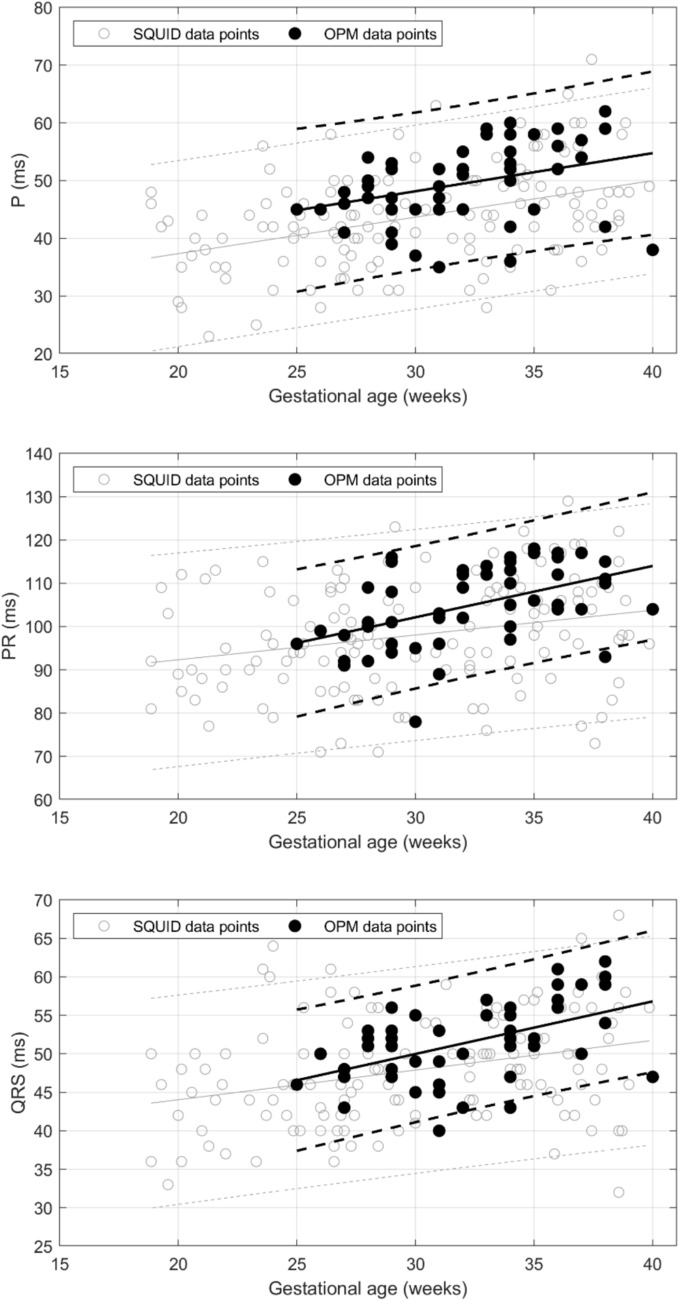

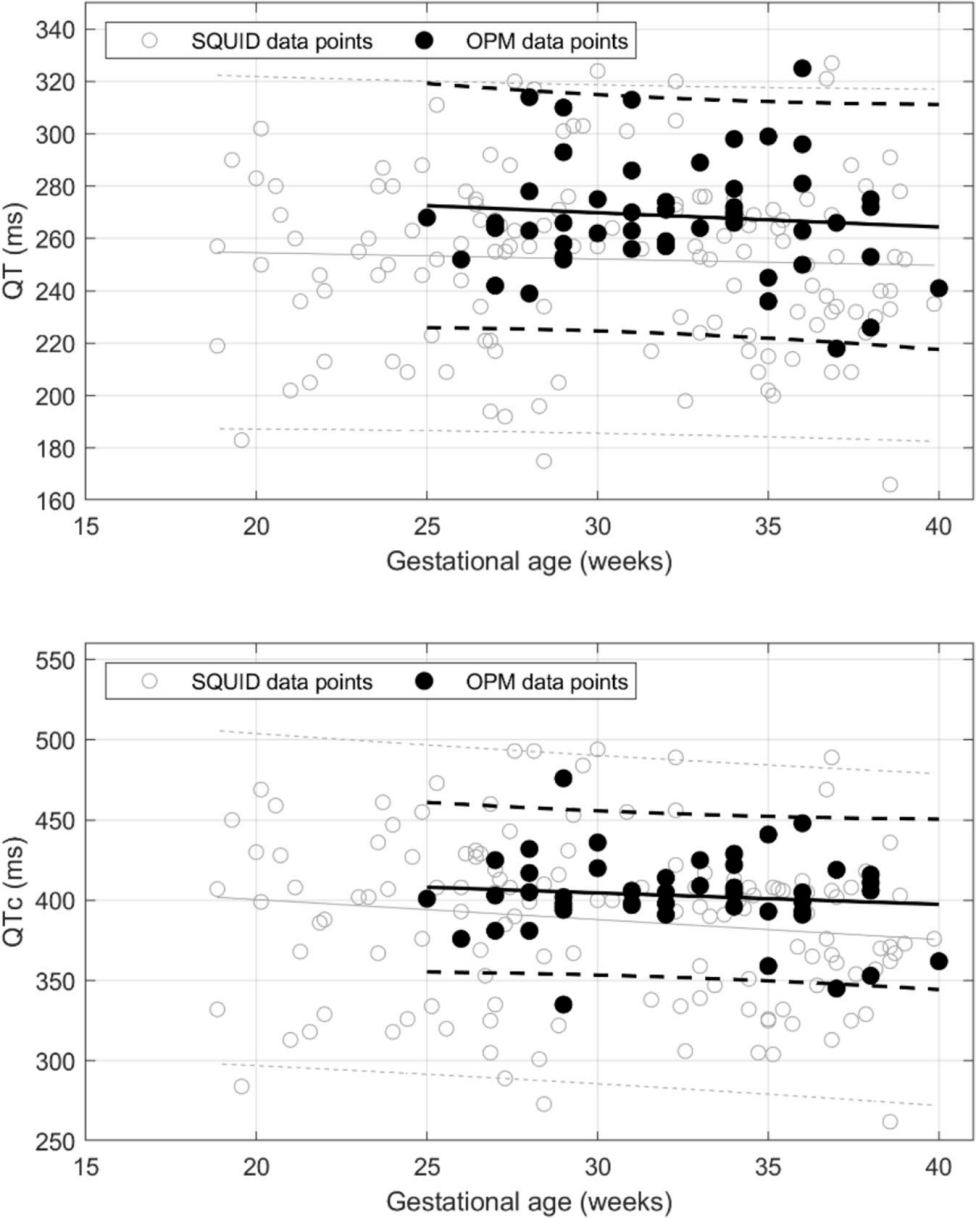
Scatter plots of fetal cardiac time intervals versus gestational age in healthy pregnancies: **a** P-wave duration, **b** PR interval, **c** QRS duration, **d** QT interval, **e** QTc interval. Black solid lines show the linear regression fits, and dashed lines denote the 5% and 95% prediction intervals. For comparison, the data of Strand et al.. is shown as gray open circles. Gray solid lines show the linear regression fits, and gray dotted lines denote the 5% and 95% prediction intervals.

Scatter plots of cardiac waveform intervals versus gestational age are shown in Fig. 2, along with linear regression lines and prediction intervals. For comparison, corresponding data from a SQUID study by Strand et al. are overlaid on each plot. Our data points lie within the prediction intervals of Strand et al., demonstrating concordance between the two studies. The results of the linear regression analysis are summarized in Table 1 and show that P-wave duration, PR interval, and QRS duration increased significantly with gestational age, whereas the QT interval, QTc, and RR did not. Except for the RR interval, Strand et al. reported the same findings. U–waves were seen in 17% of the subjects.

**Table 1:** Linear regression analysis of OPM and SQUID cardiac intervals (from Strand et al.)

Variable	OPM (n = 52)			SQUID (n = 132)		
	Mean ± SD	Linear regression		Mean ± SD	Linear regression	
		*P*-value	*R*^2^		*P*-value	*R*^2^
GA (weeks)	32 ± 3.7	–	–	30 ± 5.8	–	–
P wave (ms)	49.7 ± 7.1	0.01	0.12	43.8 ± 8.8	< 0.01	0.17
PR interval (ms)	104.9 ± 9.1	< 0.01	0.23	98.1 ± 12.7	< 0.01	0.07
QRS complex (ms)	51.6 ± 5	< 0.01	0.25	47.9 ± 7.1	< 0.01	0.10
QT interval (ms)	268.6 ± 22	0.52	0.01	252.1 ± 33.4	0.64	0.002
QTc interval (ms)	402.9 ± 25	0.46	0.01	387.8 ± 51.8	0.11	0.02
RR (ms)	426.6 ± 25.4	0.51	0.01	422.6 ± 27.1	< 0.01	0.13

Table 2 compares our fMCG cardiac time intervals with those reported in prior studies using superconducting quantum interference devices (SQUIDs). The most recent results are from the 2019 study of Strand et al. As shown in Table 2 and Fig. 2, our results are consistent with theirs. The other studies are much older but most still show good consistency with our results.

**Table 2:** Fetal cardiac time intervals of our OPM device compared to SQUID fMCG studies. Adapted from Kiefer-Schmidt et al. [22]

CTIs (ms)	GA (weeks)	fMCG OPM		fMCG SQUID						
		This study	Strand et al. [14]	Comani et al.[15]	Lowery et al.[16]	Van Leeu wen et al.[17]	Kähler et al.[18]	Stinstra et al.[19]	Horigome et al.[20]	Quinn et al.[21]
Pwave	≤ 24			68		37–47	47	42–56		
	25–26	46								
	27–28	48								
	29–30	45								
	31–33	50		70	61	56–59				
	34–35	52								
	36–37	56								
	> 37	52			58	65–72	53	98–111		71
PR interval	≤ 24		90	106		96–104	55			
	25–26	98	114							
	27–28	98	92							
	29–30	101								
	31–33	105	124	111		112–113				89
	34–35	110	106							
	36–37	111								
	> 37	107				109–113	59			43
QRS complex	≤ 24		43	54		37–41	36		41	
	25–26	48	41							
	27–28	50	45							
	29–30	51								
	31–33	49	49	57	56	48–50		43–49		47
	34–35	52	43							
	36–37	57								
	> 37	57			55	53–58	48		54	157
QT interval	≤ 24						198		223	
	25–26	260								
	27–28	267								
	29–30	271								
	31–33	273						227–255		196
	34–35	271								
	36–37	271								
	> 37	254					244		247	
QTc interval	≤ 24		414	413					333	
	25–26	389	362							
	27–28	407	371							
	29–30	408								
	31–33	405	450	432				350–400		
	34–35	406	405							
	36–37	400								
	> 37	390							393	

**Fig. 3:**
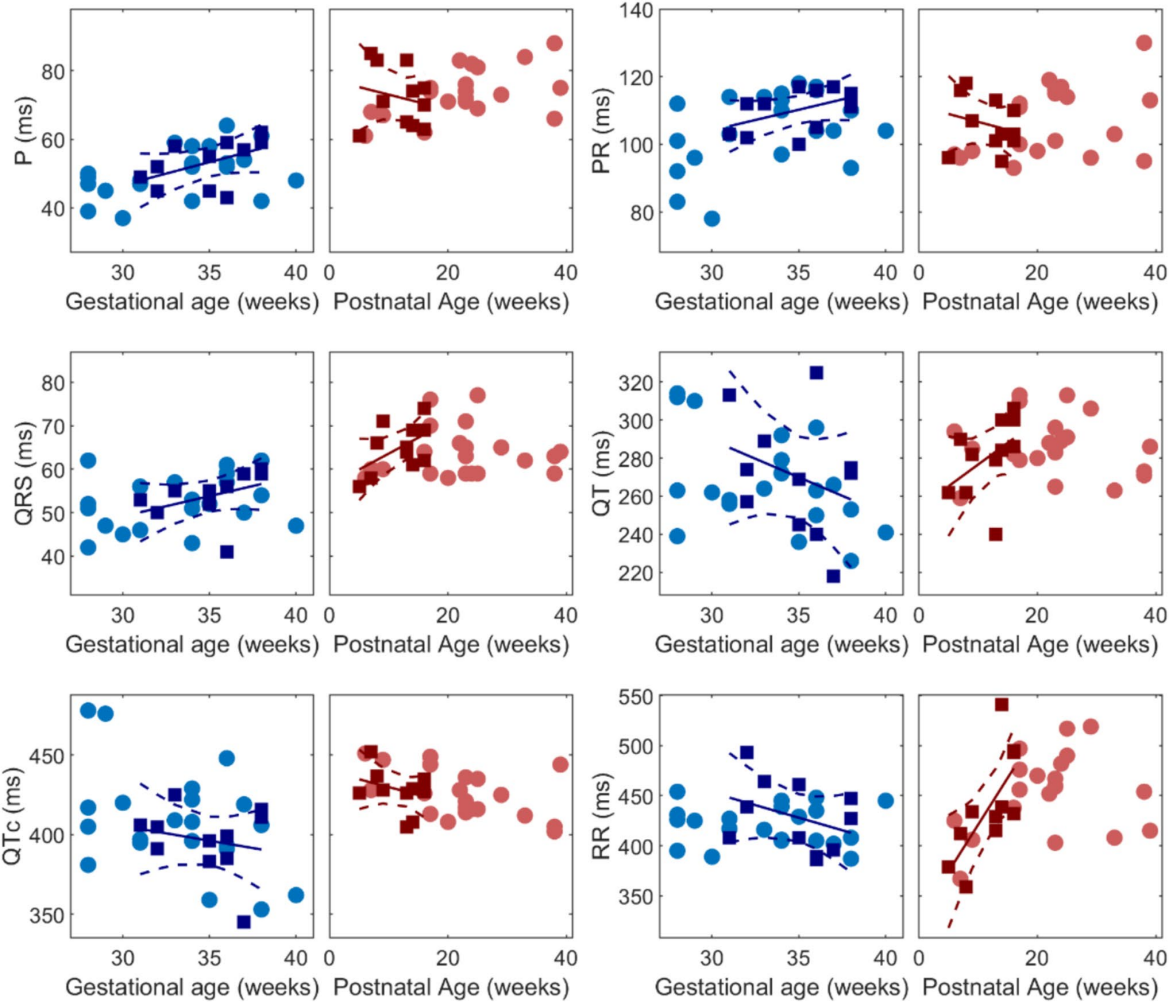
Scatter plots of cardiac time intervals in subjects with both prenatal and postnatal measurements (*n* = 32). The blue and red squares denote the perinatal cohort (*n* = 11), defined here as the subset of subjects studied prenatally after 30 weeks’ gestation and postnatally prior to 17 weeks of age. Linear regression fits to the data from the perinatal cohort are shown as solid lines, and the confidence intervals are denoted by dashed lines. The intervals did not show a statistically significant dependence on gestational age or postnatal age, except for the postnatal RR interval

Postnatal ECG measurements are shown in Fig. 3. Of the 33 recordings, only 1 was from an infant born at < 37 weeks’ gestation; the others were from term infants. One outlier with anomalous values for multiple cardiac intervals was excluded from the analysis. Eleven subjects were studied by fMCG after 30 weeks’ gestation and by ECG within 17 weeks of birth. Statistical comparisons of these paired measurements are summarized in Table 3. The P-wave duration, QRS duration, and QTc increased significantly after birth, but the PR and QT intervals did not.

**Table 3:** Comparison of fMCG and postnatal ECG cardiac time intervals

	All subjects (n = 32)		Perinatal (n = 11)		*P*-value
	fMCG	ECG	fMCG	ECG	
Age (weeks)	34 ± 3.4	19 ± 9.4	35 ± 2.6	11.82 ± 4.09	-
P wave (ms)	51.3 ± 7	73.1 ± 7.5	53.1 ± 6.7	72.2 ± 8.6	< 0.01*
PR interval (ms)	106.4 ± 10.1	106.3 ± 9.3	110 ± 6.4	105.7 ± 7.7	0.08
QRS complex (ms)	52.8 ± 5.8	64.1 ± 5.6	53.6 ± 5.4	65 ± 5.5	< 0.01*
QT interval (ms)	268.8 ± 26.8	284.4 ± 17	270.6 ± 31.1	281 ± 19.7	0.36
QTc interval (ms)	403.9 ± 29.4	426.4 ± 14.3	396.5 ± 21.4	428 ± 13	< 0.01*
RR (ms)	423.5 ± 25.8	448.2 ± 43.9	428.9 ± 34.9	438.9 ± 52.9	0.50

The means and standard deviations are shown for all subjects (*n* = 32) with both prenatal and postnatal measurements and for the perinatal cohort (*n* = 11), defined here as the subset of subjects studied prenatally after 30 weeks’ gestation and postnatally prior to 17 weeks of age. Data from the perinatal cohort were analyzed using a paired t-test and the corresponding P-values are shown in the last column. Statistically significance results are indicated by an asterisk

## Discussion

To our knowledge, this is the first large data set of healthy fetuses measured by OPM-based fMCG and compared to postnatal ECG. The main findings were that 1) the prenatal data were consistent with normative values obtained previously using SQUID devices, and 2) in the cohort studied shortly before and after birth, P-wave duration, QRS duration, and QTc increased after birth, while the PR and QT intervals did not. These results are discussed further below.

Normative fMCG interval data has been published by many groups [15–20, 22, 23]. The largest such study, performed by Stinstra and co-workers [15] in 2002, involved five centers and 582 normal fetuses. A confounding factor, however, was that this and other early studies were performed using different fMCG systems and methodologies. The last 20 years have seen significant improvements in instrumentation and signal analysis methods. In addition, more recent studies have usually been interpreted by fetal cardiologists rather than basic scientists, which likely resulted in more accurate measurements. For this reason, we established concordance by comparing our data to that from a large, recent single-center study by Strand et al. [11], which involved a fetal cardiologist and applied the same analysis methods as used in our study. In a subsequent study of subjects with uncomplicated pregnancies and pregnancies complicated by fetal arrhythmia and other conditions, Strand et al. [7] demonstrated for the first time that OPMs were comparable to SQUIDs for fMCG. In addition to signal-to-noise ratio, waveform interval measurements were used to characterize and compare data from the OPM and SQUID systems. The measurements showed good agreement; however, a limitation was the small number of healthy fetuses (*n* = 6) compared to our cohort.

Characterizing normal subject data is essential for clinical application. The establishment of 95% prediction intervals in healthy fetuses is especially important for detection of conduction abnormalities, such as PR prolongation in AV block and QRS prolongation in ventricular rhythm, and repolarization abnormalities, such as QTc prolongation in long QT syndrome. Notably, U-waves were seen in 9 of 52 (17.3%) of our subjects and 11% of subjects studied by Strand et al. The frequent occurrence of U-wave is important to recognize because its presence can confound the diagnosis of long QT syndrome.

Postnatal changes in cardiac intervals are to be expected but have not been shown previously. The circulation undergoes dramatic change at birth. The sudden onset of pulmonary flow significantly increases the effective cardiac output, leading to increased filling and subsequent hypertrophy. Consistent with these structural adaptations, the P-wave and QRS durations increased abruptly at birth. QTc also increased; however, this may be artifactual because the QT interval did not increase, implying that the change reflects alterations in heart rate rather than repolarization. In contrast, the PR interval did not change. This suggests that the conduction pathways adapt to chamber enlargement to maintain AV conduction time.

Currently, the device in our center is only used for basic research. Once OPM devices are approved for clinical application, the ultimate measure of success is the ability of the pediatric cardiologist or perinatologist to assess the cardiac rhythm precisely and make an accurate diagnosis before birth. This is important for possible treatment options pre- and postnatally. The excellent performance of SQUID systems has been corroborated by their efficacy for evaluation of fetuses with serious arrhythmia [11]. The concordance of our results with prior studies demonstrates that OPM-based fMCG is equivalent to SQUID-based fMCG, despite fundamental differences in sensor technology. We speculate that OPMs might expand these efforts in the future as they can provide comparable results at substantially lower cost.

## Conclusion

To our knowledge, this is the first study of healthy fetuses comparing prenatal measurements of an OPM device with the postnatal ECG. The prenatal data of our OPM device was comparable to normative values of previously used SQUID devices, and the data recorded in near-term fetuses was comparable to infant ECG. Interestingly, there is a pronounced postnatal increase in P-wave and QRS duration at the time of birth which would be expected but has not been shown so far. OPM fMCG has high potential for clinical evaluation of fetal cardiac time intervals and diagnosis of fetal arrhythmia and conduction disease.

## Data Availability

An anonymized data set can be made available upon request. Access is only granted to academic institutions and after signing a data share agreement.

## References

[1] Wacker-Gussmann A, Strasburger JF, Wakai RT (2022) Contribution of fetal magnetocardiography to diagnosis, risk assessment, and treatment of fetal arrhythmia. J Am Heart Assoc 11(15):e025224 35904205 10.1161/JAHA.121.025224 PMC9375504

[2] Peters C, Wacker-Gussmann A, Strasburger JF, Cuneo BF, Gotteiner NL, Gulecyuz M et al (2015) Electrophysiologic features of fetal ventricular aneurysms and diverticula. Prenat Diagn 35(2):129–136 25284224 10.1002/pd.4501 PMC4319987

[3] Wacker-Gussmann A, Strasburger JF, Srinivasan S, Cuneo BF, Lutter W, Wakai RT (2016) Fetal atrial flutter: electrophysiology and associations with rhythms involving an accessory pathway. J Am Heart Assoc 5(6):e003673 27302699 10.1161/JAHA.116.003673 PMC4937288

[4] Wacker-Gussmann A, Strasburger JF, Wakai RT (2022) Fetal magnetocardiography alters diagnosis and management in fetal congenital heart disease and cardiomyopathy. JACC Clin Electrophysiol 8(9):1159–1161 36137723 10.1016/j.jacep.2022.04.012

[5] Wacker-Gussmann A, Tardelli G, Cuneo BF, Strasburger JF, Wakai RT (2025) Fetal conduction disease and arrhythmia in Ebstein’s anomaly and tricuspid valve dysplasia assessed by fetal magnetocardiography. J Am Heart Assoc 14(15):e043614 40728160 10.1161/JAHA.125.043614 PMC12449953

[6] Joglar JA, Kapa S, Saarel EV, Dubin AM, Gorenek B, Hameed AB et al (2023) 2023 HRS expert consensus statement on the management of arrhythmias during pregnancy. Heart Rhythm 20(10):e175-264 37211147 10.1016/j.hrthm.2023.05.017

[7] Donofrio MT, Moon-Grady AJ, Hornberger LK, Copel JA, Sklansky MS, Abuhamad A et al (2014) Diagnosis and treatment of fetal cardiac disease. Circulation 129(21):2183–2242 24763516 10.1161/01.cir.0000437597.44550.5d

[8] Shah VK, Wakai RT (2013) A compact, high performance atomic magnetometer for biomedical applications. Phys Med Biol 58(22):8153–8161 24200837 10.1088/0031-9155/58/22/8153 PMC3971838

[9] Taggart NW, Haglund CM, Tester DJ, Ackerman MJ (2007) Diagnostic miscues in congenital long-QT syndrome. Circulation 115(20):2613–2620 17502575 10.1161/CIRCULATIONAHA.106.661082

[10] Escalona-Vargas D, Bolin EH, Lowery CL, Siegel ER, Eswaran H (2021) Recording and quantifying fetal magnetocardiography signals using a flexible array of optically-pumped magnetometers. Physiol Meas 41(12):125003 33086201 10.1088/1361-6579/abc353 PMC7875519

[11] Strand SA, Strasburger JF, Wakai RT (2019) Fetal magnetocardiogram waveform characteristics. Physiol Meas 40(3):035002 30802886 10.1088/1361-6579/ab0a2c PMC6449176

[12] Yu S, Wakai RT (2011) Maternal MCG interference cancellation using splined independent component subtraction. IEEE Trans Biomed Eng 58(10):2835–2843 21712157 10.1109/TBME.2011.2160635 PMC3179557

[13] Lutter WJ, Wakai RT (2011) Indices and detectors for fetal MCG actography. IEEE Trans Biomed Eng 58(6):1874–1880 21427015 10.1109/TBME.2011.2131141 PMC3098322

[14] Strand S, Lutter W, Strasburger JF, Shah V, Baffa O, Wakai RT (2019) Low-cost fetal magnetocardiography: a comparison of superconducting quantum interference device and optically pumped magnetometers. J Am Heart Assoc 8(16):e013436 31394997 10.1161/JAHA.119.013436 PMC6759914

[15] Comani S, Liberati M, Mantini D, Merlino B, Alleva G, Gabriele E et al (2005) Beat-to-beat estimate of fetal cardiac time intervals using magnetocardiography: longitudinal charts of normality ranges and individual trends. Acta Obstet Gynecol Scand 84(12):1175–1180 16305704 10.1111/j.0001-6349.2005.00855.x

[16] Lowery CL, Campbell JQ, Wilson JD, Murphy P, Preissl H, Malak SF et al (2003) Noninvasive antepartum recording of fetal S-T segment with a newly developed 151-channel magnetic sensor system. Am J Obstet Gynecol 188(6):1491–1496 (**discussion 1496-1497**) 12824983 10.1067/mob.2003.367

[17] Van Leeuwen P, Lange S, Klein A, Geue D, Grönemeyer DH (2004) Dependency of magnetocardiographically determined fetal cardiac time intervals on gestational age, gender and postnatal biometrics in healthy pregnancies. BMC Pregnancy Childbirth 4(1):6 15061871 10.1186/1471-2393-4-6 PMC411040

[18] Kähler C, Schleussner E, Grimm B, Schneider A, Schneider U, Nowak H et al (2002) Fetal magnetocardiography: development of the fetal cardiac time intervals. Prenat Diagn 22(5):408–414 12001197 10.1002/pd.322

[19] Stinstra J, Golbach E, van Leeuwen P, Lange S, Menendez T, Moshage W et al (2002) Multicentre study of fetal cardiac time intervals using magnetocardiography. BJOG 109(11):1235–1243 12452461 10.1046/j.1471-0528.2002.01057.x

[20] Horigome H, Takahashi MI, Asaka M, Shigemitsu S, Kandori A, Tsukada K (2000) Magnetocardiographic determination of the developmental changes in PQ, QRS and QT intervals in the foetus. Acta Paediatr 89(1):64–67 10677060 10.1080/080352500750029086

[21] Quinn A, Weir A, Shahani U, Bain R, Maas P, Donaldson G (1994) Antenatal fetal magnetocardiography: a new method for fetal surveillance? BJOG 101(10):866–870 10.1111/j.1471-0528.1994.tb13547.x 7999688

[22] Kiefer-Schmidt I, Lim M, Wacker-Gussmann A, Ortiz E, Abele H, Kagan KO et al (2012) Fetal magnetocardiography (fMCG): moving forward in the establishment of clinical reference data by advanced biomagnetic instrumentation and analysis. J Perinat Med 40(3):277–286 22505507 10.1515/jpm.2011.139

[23] Stingl K, Paulsen H, Weiss M, Preissl H, Abele H, Goelz R et al (2013) Development and application of an automated extraction algorithm for fetal magnetocardiography - normal data and arrhythmia detection. J Perinat Med 41(6):725–734 23828424 10.1515/jpm-2013-0031

